# Child malaria vaccine uptake in Ghana: Factors influencing parents’ willingness to allow vaccination of their children under five (5) years

**DOI:** 10.1371/journal.pone.0296934

**Published:** 2024-01-19

**Authors:** Sulemana Ansumah Saaka, Kamaldeen Mohammed, Cornelius K. A. Pienaah, Isaac Luginaah

**Affiliations:** Department of Geography and Environment, Faculty of Social Science, University of Western, London, Ontario, Canada; Freelance Consultant, Myanmar, MYANMAR

## Abstract

**Background:**

Malaria is a substantial health burden in Ghana, particularly among children. Despite the availability of malaria vaccines, uptake remains low. Notwithstanding, there is a paucity of nationally representative studies on the factors driving hesitance towards the new malaria vaccine. In response, this study, guided by the Theory of Planned Behaviors (TPB), seeks to understand the determinants of child malaria vaccine uptake in Ghana to inform strategies for improving coverage.

**Materials and methods:**

We employed multiple regression model to examine the association between maternal awareness, socioeconomic status, ethnicity, geographical location, and vaccine uptake using data from the 2019 Ghana Malaria Indicator Survey (MIS).

**Results:**

Maternal awareness of vaccine (OR = 2.200; P<0.01) significantly predicted higher likelihood of vaccine uptake. Household wealth was associated with child vaccination as parents in middle-income households (OR = 9.342; P<0.01), and those in poorest households (OR = 9.409; P<0.05) recorded higher likelihood of allowing their children to be vaccinated. With regards to ethnicity, parents from the Mande ethnic group (OR = 0.106; P<0.05) were less likely to allow their children to be vaccinated when compared to parents from the Akan ethnic group. Knowing that malaria is covered by National Health Insurance (OR = 2.407; P<0.05) was associated with higher likelihood of allowing child vaccination compared to not knowing. More so, geographical variations were observed as parents who lived in rural areas (OR = 0.254; P<0.05) were significantly less likely to allow vaccination of their children compared to those in urban areas.

**Conclusions:**

Enhancing awareness through education campaigns can improve child malaria vaccine coverage. Observing socioeconomic disparities in uptake and ensuring equitable access to vaccines are vital. Tailored strategies considering ethnic background and geographical location, can as well enhance acceptance of the vaccine. This study provides valuable insights for developing effective strategies to reduce the burden of malaria in children and improve coverage of uptake. This study underscores the need to improve parental awareness and the relevance of the vaccine in preventing child mortality.

## Introduction

Malaria, a disease caused by the Plasmodium parasite, remains a substantial global health challenge [1, 2]. According to the World Health Organization (WHO) malaria report, there were 229 million cases and 409,000 deaths in 2019 alone, with most cases occurring in sub-Saharan Africa [3]. Children under five are particularly vulnerable, accounting for the highest-burden of malaria-related morbidity and mortality [4, 5].

As part of efforts to tackle this important public health crisis, different control strategies have been implemented including the development of malaria vaccines. Vaccines have proven to be effective in preventing infectious diseases and reducing associated morbidity and mortality [6]. The RTS,S/AS01 malaria vaccine, also known as Mosquirix, is currently the most advanced malaria vaccine and has shown efficacy in clinical trials [7]. Ghana was one of the first three African countries to partake in the malaria vaccine program in 2019 which targeted children under five years of age [8]. However, despite the availability of the vaccines, uptake remains a challenge in many affected regions, hindering efforts to reduce the burden of the disease.

Across Sub-Sahara Africa (SSA), Ghana is among the countries with high rates of malaria infection [9]. The Ghana Health Service reported that malaria accounts for a significant proportion of outpatient visits, hospital admissions, and child deaths in the country [10]. As part of efforts to combat the disease, Ghana government implemented various interventions, including the distribution of insecticide-treated bed nets and the provision of prompt and effective treatment [11]. The government of Ghana is also the first to endorse the Oxford base malaria vaccine as part of an effort to alleviate the burden of malaria in the country.

While these interventions such as insecticide-treated bed nets have contributed to the reduction of malaria cases, the uptake of malaria vaccines in Ghana remains low, especially among children [12]. Understanding the factors that influence child malaria vaccine uptake is crucial for designing effective strategies to improve coverage and to protect vulnerable populations such as children [13]. This study seeks to contribute to the existing knowledge by examining the determinants of child malaria vaccine uptake in Ghana. The findings will provide valuable insights for policymakers, healthcare providers, and researchers working towards reducing the burden of malaria in the country.

### Theoretical underpinning

The study draws its theoretical conceptualization from the theory of planned behaviors (TPB) [14]. Theory of planned behaviors is one of the most robust frameworks for explanation of health-related behaviors [15]. TPB posits that three factor—*subjective norms (i*.*e*., the perceived social pressure to perform or not to perform the behavior*)*, *attitude towards the behavior* (*i*.*e*., the extent to which the individual has a favorable or unfavorable judgement of the act or behavior in question), *and perceived behavioral control* (*i*.*e*., the perceived ease or difficulty of performing the given behavior, informed mostly by past experiences and anticipated impediments) interact to shape the behavioral intentions of an individual and influence the individual’s willingness to accept an idea or perform a given behavior. For instance, the perceived efficacy and possible side effects of vaccines, misinformation, or public mistrust in vaccines, as well as knowledge on cost of vaccination are all factors that may either induce favorable or unfavorable judgement towards vaccination exercises [16]. Similarly, family pressure, peer influence, media influence, community expectations or collective responsibility, workplace requirement, extensive information search etc., are forms of subjective norms that play a significant role in shaping the individual’s decision to get vaccinated [17].

Fan et al. after employing theory of planned behaviors in the explanation of intentions of university students in China to vaccinate against COVID-19, discovered that past vaccination was positively associated with intention to get vaccinated [15]. “Intentions are assumed to capture the motivational factors that influence a behavior; they are indications of how hard people are willing to try, of how much of an effort they are planning to exert, in order to perform the behavior” [14]. Behavioral intentions, however, can only translate into actual behavior if the behavior in question is under volitional control (i.e., only if the person can decide at will to perform or not perform the behavior), according to TPB. In the case of child vaccine uptake for instance, parents have volitional control, where their willingness to allow vaccination of their children is dependent on their perceived ease or difficulty regarding vaccination, their judgement of the vaccine, as well as their perceived social pressure to or not to vaccinate the child. Moreover, the performance and success of most health behaviors depends on nonmotivational factors such as the availability of necessary opportunities and resources (e.g., knowledge, time, money, skills) [14]. For instance, knowledge of the vaccine, the perceived risk of child infection, distance to the vaccination center, waiting time spent on queues, and even the cost of transport to the center of vaccination, may collectively interact to determine uptake irrespective of availability of vaccines at designated centers. Thus, with the required opportunities and resources, and intends to perform the behavior, the propensity of success may be high, all other things equal.

In certain contexts, not only perceived social pressures, but also personal feelings of moral obligation are key to the given behavior [14]. Such moral obligations may influence intentions, in correspondence with attitudes, subjective social norms and perceptions of behavioral control. For example, in most Ghanaian communities, especially in patriarchal communities of Northern Ghana, household heads have the moral obligation to take major household decisions [18], which have bearing on the behavior of members of the household at large.

**Fig 1:**
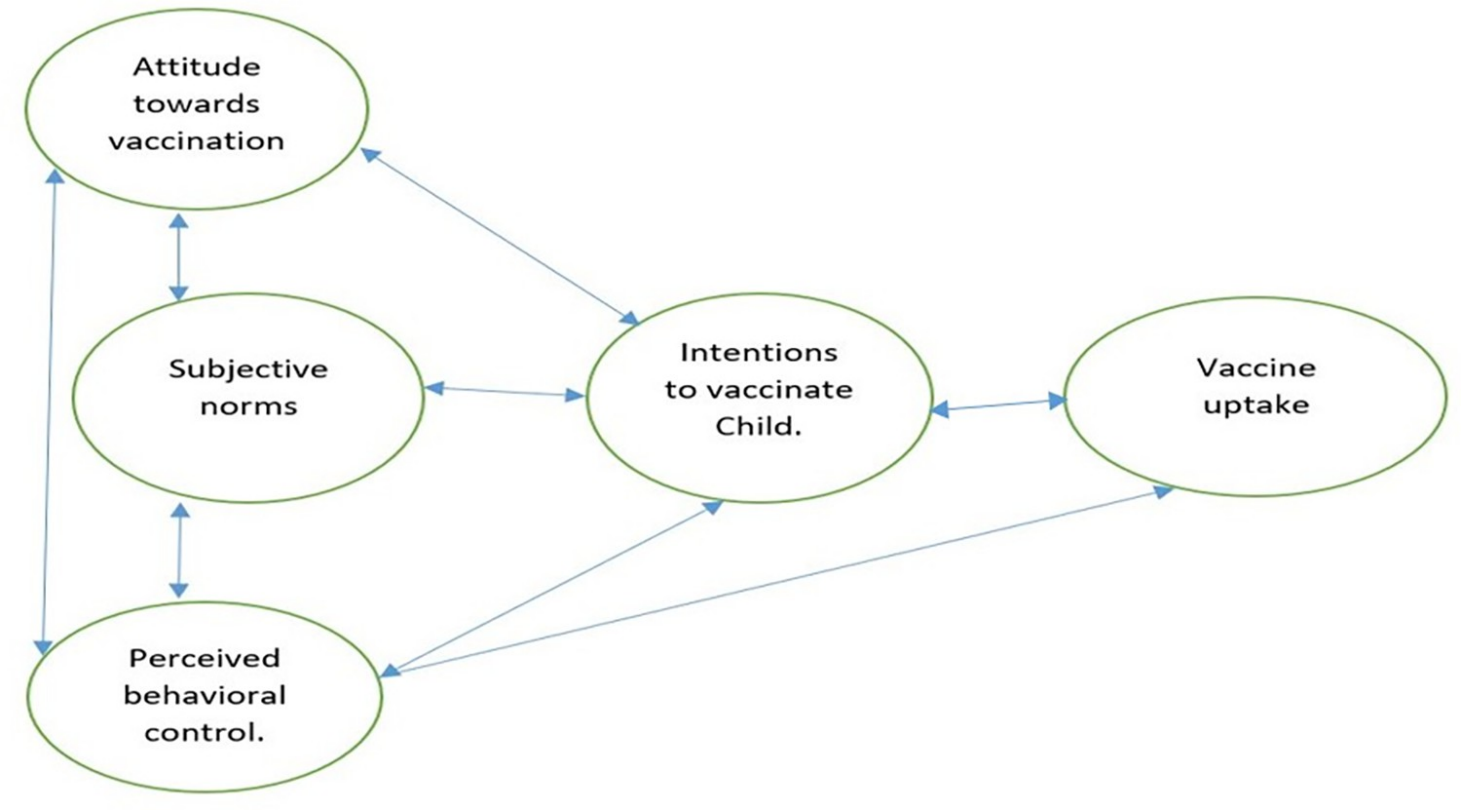
Modified diagrammatic representation of the theory of planned behavior. Source: [14].

TPB emphasizes the need to understand that these factors (i.e., subjective norms, attitudes, and perceived behavioral control) are not deterministic, but rather, they provide a framework for understanding the underlying influences on health behaviors and actions. TPB has been widely adopted and utilized in the field of health sciences to study health-related behaviors including behaviors towards vaccine uptake [15, 19, 20] with *attitude toward the action and perceived behavioral control* serving as significant variables responsible for possible variation in intentions behind the behavior in question [21]. Vaccines are one of the contested health interventions, for varied reasons including issues of trust and efficacy [22, 23]. Thus, adopting this theory in our study, we explored the factors influencing parents’ willingness to allow vaccination of children under five years of age against malaria, following the lunch of the new malaria vaccination program in Ghana in 2019. Fig 1 is a diagrammatic representation of TPB.

## Materials and methods

### Study context

With an area of approximately 238,535 square kilometers, Ghana is bordered by Côte d’Ivoire, Burkina Faso, Togo, and the Atlantic Ocean [24]. It is a West African country known for its cultural heritage, vibrant markets, and historical landmarks (GSS, 2013). The population of Ghana is 31 million people, with the capital city being Accra (GSS, 2020). Other major cities include Kumasi, Tamale, and Sekondi-Takoradi. The country is home to diverse ethnicity, with Akan, Mole-Dagbon, Ewe, and Ga-Adangbe being the most popular ethnic groups. While various indigenous languages are spoken, English is the official language (GSS, 2013).

Ghana’s economy relies on agriculture, mining, and services, with commodities such as cocoa, gold, oil, and timber being major exports [24]. The country has experienced considerable economic growth and progress in poverty reduction and human development [24] but the economy declined in recent with many experiencing hardships at the household level [25]. Ghana faces significant health burden, including malaria, which is endemic in the country [26]. Malaria is a substantial public health burden in Ghana, particularly in rural areas where there is high transmission rates [9, 26]. Control and prevention efforts involve the distribution of insecticide-treated bed nets, indoor residual spraying, and prompt diagnosis and treatment [26].

### Data collection

This study draws data from the 2019 Ghana Malaria Indicator Survey (MIS), conducted by Ghana Statistical Service (GSS) from September 2019 to November 2019. The 2019 Ghana Malaria Indicator Survey (2019 GMIS) is the second MIS conducted in Ghana, after the 2016 GMIS. The 2019 GMIS used a nationally representative sample of 200 clusters and about 6,000 selected households. Protocols for the 2019 GMIS was approved by the Ghana Health Service Ethical Review Committee and International Coaching Federation’s (ICF) Institutional Review Board. Prior to the collection of blood samples for malaria and anaemia testing, informed consent was requested from parents or guardians of children. Participation in the survey was voluntary hence Informed consent was sought verbally from eligible respondents prior to the administration of questionnaire [27]. All data and other information collected were confidential.

### Sampling and selection

Although the government of Ghana created six additional regions in 2019, during the survey design, the new administrative boundaries were not available. Therefore, the sampling frame for the 2019 GMIS was based on the Ghana Population and Housing Census (PHC) conducted in 2010 by the Ghana Statistical Service (GSS). As part of the 2010 PHC, Ghana was administratively divided into 10 geographical regions. The sampling frame contains information about the location of enumeration areas (EAs), type of residence (urban or rural), the estimated number of residential households, and the estimated population. The 2019 GMIS sample was stratified and selected from the sampling frame in two stages. Each region was divided into urban and rural areas; this yielded 20 sampling strata. In the second stage of selection, a fixed number of 30 households was selected from each cluster to make up a total sample size of 6,000 households. Replacement of non-responding households was not allowed. All women age 15–49 who were either permanent residents of the selected households or visitors who stayed in the household the night before the survey were eligible to be interviewed [26]. Details on the methods of data collection can be found at https://microdata.worldbank.org/index.php/catalog/3752

### Measures

#### Outcome variable

The outcome variable for this study is the derived from a question that asked whether parents/guardians/parents ‘*Will allow child to be vaccinated’* (0 = "No”, 1 = "Yes").

#### The explanatory variables include

Aware of malaria vaccine (0 = No, 1 = Yes); aware that malaria is covered by the National Health Insurance Scheme [NHIS (0 = no, 1 = yes)]; malaria program (0 = Never enrolled, 1 = Enrolled); Religion (0 = Christian, 1 = Islam, 2 = traditional/spiritualist, 3 = no religion/other); Ethnicity (1 = Akan, 2 = Mole-Dagbani, 3 = ewe, 4 = Gurma/Grusi, 5 = Ga/Dangme, 6 = Guan, 7 = Mande); wealth (0 = Richest, 1 = Richer, 2 = Middle, 3 = Poorer, 4 = Poorest); (0 = Secondary/SSS/SHS or higher, 1 = Middle/JSS/JHS, 2 = Primary, 3 = No education); Sex of household head (1 = male, 2 = female); whether mother is related to household head (0 = Not related, 1 = Related); Age bracket of mother (0 = 36–49, 1 = 15–35); Sex of child (0 = Male, 1 = Female); Age of child (0 = 2–4 year, 1 = Under 2 years); have live births between births (0 = no, 1 = Yes); where child/children lives (1 = With parent, 2 = Elsewhere); Children sleep under mosquito nets (0 = no, 1 = Yes); type of place of residence (1 = urban, 2 = rural); Regions (0 = Greater Accra, 1 = Western region, 2 = Central region, 3 = Volta region, 4 = Eastern region, 5 = Ashanti region, 6 = Brong Ahafo region, 7 = Northern region, 8 = Upper East region, and 9 = Upper West region).

#### Recoding of variables

Education variable was recoded as 3 = 0 "secondary/SSS/SHS or higher”, 2 = 1 "middle/JSS/JHS", 1 = 2 "Primary", 0 = 3 "No education" because we were contextually interested in predicting parents with lesser educational attainment in relation to those with higher educational attainment. Also, the wealth index variable was coded as 5 = 0 "richest", 4 = 1 "richer", 3 = 2 "Middle", 2 = 3 "Poorer", and 1 = 4 "Poorest" to make prediction for parents in lower wealth quantiles. Likewise, the regions variable was coded as 3 = 0 "Greater Accra", 1 = 1 "Western", 2 = 2 "central", 4 = 4 "Volta", 5 = 5 "Eastern", 6 = 6 "Ashanti", 7 = 7 "Brong Ahafo", 8 = 8 "Northern", 9 = 9 "Upper East", 10 = 10 "Upper West" to make Greater Accra the reference category while predicting other regions.

### Analytical approach

We employed both descriptive and inferential statistical analyses in this study. First, we provided a statistical description of the distribution of all the study variables. Secondly, a binary logistic regression model was used to examine the relationship between predictor variables and child malaria vaccine uptake. Given the dichotomous nature of the outcome variable, logistic regression was more appropriate. Thus, a bivariate logistic regression was employed to comprehend the relationship between each predictor variable and the outcome variable. Multiple logistic regression analysis was further employed to ascertain relationships between the selected predictor variables and child malaria vaccine uptake. Results of the regression models are reported in Odds ratios (OR). Significant Odds ratios above one (OR > 1) shows a higher likelihood of parents allowing their children to be vaccinated against malaria while Odds ratios below one (OR<1) shows a lower likelihood of parents allowing their children to be vaccinated against malaria. All statistical data analyses were performed in Stata version 18.

## Results and discussion

### Univariate analysis

From the statistics in Table 1, participants with no prior awareness of the vaccine availability and yet willing to allow vaccination of their children under 5 were more (1,599 parents) compared to those with prior awareness of the vaccine (1,211 parents) and willing to allow vaccination of their children. Also, those who were not aware and not willing to allow vaccination of their children were more (138) compared to those who were aware but not willing to allow vaccination of their children (56).

**Table 1:** A cross tabulation of vaccine awareness and willingness to allow vaccination of children.

Awareness of malaria vaccine	Willingness to allow child	to be vaccinated	Total
	No	Yes	
No	138	1,599	1,737
Yes	56	1,211	1,267
**Total**	194	2,810	3,004

Table 2 presents the results for descriptive statistics of the study variables. Majority of the parents/guardians (93.54%) expressed willingness to allow vaccination of children under five. Majority of parents (57.82%) indicated non-awareness of the vaccine. In terms of educational attainment, 27.50% had no formal education while the overwhelming majority had some form of formal education (see Table 2). Also, majority (53.03%) of the respondents were Christians. Likewise, the Akan ethnic group constituted a greater proportion (33.99%) of the participants, followed the Mole-Dagbani (32.42%) and the Ewes (12.35%). Geographically, 61.05% of the participants resided in rural areas while only 38.95% resided in urban areas. See Table 2 for details on the distribution of all independent variables included in the analysis.

**Table 2:** Descriptive statistics.

Predictor variables	Distribution (%)
**Allowed child to be vaccinated**	
No	6.46
Yes	93.54
**Aware/heard of malaria vaccine**	
No	57.82
Yes	42.18
**Education**	
SSS/SHS or Higher	16.78
Middle/JSS/JHS	34.22
Primary	21.50
No education	27.50
**Wealth**	
Richest	12.02
Richer	14.75
Middle	19.84
Poorer	21.40
Poorest	31.99
**Religion**	
Christians	53.03
Muslims	23.83
Traditionalist/Spiritualists	15.28
No religion/Other	7.86
**Ethnicity**	
Akan	33.99
Mole-Dagbani	32.42
Ewe	12.35
Gurma/Grusi	9.92
Ga/dangme	4.89
Guan	5.26
Mande	1.17
**Malaria Program**	
Enrolled	18.21
Never enrolled	81.79
**Aware Malaria is covered by NHIS**	
Yes	75.13
No	24.87
**Age of mother**	
15–35 years	78.23
36–49 years	21.77
**Children sleep under mosquito net**	
Yes	63.35
No	36.65
**Sex of child**	
Female	49.63
Male	50.37
**Age of child**	
Under 2 years	40.78
2–4 years	56.69
**Where child lives**	
With parent/mother	96.24
Lives elsewhere	3.76
**Related to household head**	
Related	92.64
Not related	7.36
**Regions**	
Greater Accra	7.92
Western	9.79
Central	9.05
Volta	8.32
Eastern	8.46
Ashanti	10.35
Brong Ahafo	9.62
Northern	16.34
Upper East	9.69
Upper West	10.45
**Place of residence**	
Urban	38.95
Rural	61.05

### Results for bivariate analysis

Table 3 shows the results for our bivariate analysis. At the bivariate level, there is significant association between the outcome variable (i.e., Willingness to vaccinate) and a several predictor variables. Parents with awareness of the vaccine (OR = 1.866; P<0.001), and those who knew that malaria is covered by national health insurance (OR = 1.533; P<0.01) were significantly more likely to allow their children to be vaccinated than those who were not.

**Table 3:** Logistic regression analysis of the predictors of child malaria vaccine uptake.

Variable	Bivariate	CI (95%)	Multivariate	CI (95%)
	OR(SE)		OR(SE)	
**Aware of malaria vaccine** (Ref: No)				
Yes	1.866(0.304) ***	1.356 2.568	2.200(0.827) **	1.052 4.599
**Education** (Ref: SSS/SHS or Higher)				
Middle/JSS/JHS	1.362(0.272)	0.921 2.015	1.615(0.887)	0.550 4.742
Primary	1.568(0.358) *	1.001 2.456	1.175(0.761)	0.330 4.182
No education	1.830(0.407) *	1.183 2.830	1.247(0.877)	0.314 4.954
**Wealth** (Ref: Richest)				
Richer	1.879(0.474) *	1.145 3.084	3.626(2.542)	0.9180 14.328
Middle	2.574(0.647) ***	1.572 4.213	9.342(7.640) *	1.880 46.408
Poorer	2.156(0.508) ***	1.35 3.423	4.530 (3.565)	0.968 21.189
Poorest	2.088(0.445) ***	1.374 3.173	9.409 (8.346) *	1.654 53.530
**Religion** (Christians)				
Islam	1.528(0.303) *	1.035 2.254	1.459(0.912)	0.428 4.968
Traditional/spiritualist	1.307(0.292)	0.843 2.027	1.141(0.579)	0.422 3.085
no religion/other	1.011(0.272)	0.596 1.716	0.682(0.422)	0.202 2.297
**Ethnicity** (Ref: Akan)				
Mole-Dagbani	1.166(0.235)	0.784 1.733	1.895(1.775)	0.302 11.890
Ewe	0.439(0.092) ***	0.290 0.664	0.523(0.312)	0.162 1.688
Gurma/Grusi	0.868(0.237)	0.507 1.484	0.666(0.530)	0.140 3.167
Ga/dangme	0.452(0.131) *	0.255 0.800	0.823(0.602)	0.196 3.454
Guan	2.276(1.193)	0.814 6.363	1.109(1.117)	0.153 7.992
Mande	0.285(0.133)		0.106(0.110) *	0.013 0.809
**Past enrollment in malaria Program** (Ref: Never enrolled)				
Ever enrolled	1.061(0.221)	0.705 1.597	1.086(1.049)	0.163 7.214
**Aware Malaria is covered by NHIS** (Ref: No)				
Yes	1.533(0.243) **	1.122 2.093	2.407(0.887) *	1.169 4.957
**Age of mother** (Ref:36–49 years)				0.185 1.459
15–35 years	0.779(0.149)	0.534 1.135	0.520(0.273)	
**Children sleep under mosquito net** (Ref: No)				
Yes	1.201(0.181)	0.893 1.616	1.903(0.807)	0.828 4.370
**Livebirths between births** (Ref: No)				
Yes	0.580(0.268)	0.234 1.438	0.410(0.217)	0.145 1.157
**Sex of child** (Ref: Male)				
Female	0.963(0.142)	0.719 1.288	1.124(0.394)	0.564 2.238
**Age of child** (Ref:2–4 years)				
Under 2 years of age	1.025(0.156)	0.760 1.383	3.706(4.051)	0.435 31.571
**Where child lives** (Ref: With parent)				
Lives elsewhere	0.902(0.338)	0.433 1.880	0.833(0.692)	0.163 4.252
**Sex of household head** (Ref: Male)				
Female	1.025(0.170)	0.740 1.421	0.977(0.418)	0.422 2.263
**Whether mother is related to household head** (Ref: Not related)				
Related	0.385(0.1620)	0.168 .878	0.248(0.267)	0.029 2.055
**Region of residence** (Ref: Greater Accra)				
Western	1.803(0.542) *	1.000 3.253	0.788(0.663)	0.151 4.106
Central	2.557(0.865) *	1.317 4.964	1.140(1.071)	0.180 7.188
Volta	1.306(0.381)	0.736 2.316	0.799(0.701)	0.143 4.458
Eastern	1.463(0.436)	0.815 2.626	0.697(0.563)	0.143 3.401
Ashanti	7.053(3.226) ***	2.877 17.287	8.717(11.234)	0.697 108.996
Brong Ahafo	2.220(0.708) *	1.188 4.148	0.480(0.412)	0.089 2.587
Northern	3.646(1.135) ***	1.980 6.712	1.714(1.899)	0.195 15.033
Upper East	2.104 (0.660) *	1.137 3.892	0.594(0.860)	0.034 10.151
Upper West	1.604(0.461) *	0.912 2.818	0.189(0.271)	0.0115 3.128
**Type of place of residence** (Ref: Urban)				
Rural	0.898(0.138)	0.664 1.215	0.254(0.147) *	0.081 0.795

*P<0.05

**P<0.01

***P<0.001; Odd Ratio (OR), Standard Error (SE), Confidence Interval (CI)

Also, the educational attainment of parents significantly predicted child malaria vaccine uptake whereby parents with primary education (OR = 1.568 P<0.05) and those with no formal education (OR = 1.830; P<0.05) were significantly more likely to allow vaccination of children under their care compared to those with higher educational attainment. With regards to wealth, parents from the richer (OR = 1.879 P<0.05), middle-income (OR = 2.574; P<0.001), Poor (OR = 2.156; P<0.001), and poorest households (OR = 2.088; P<0.001) were all significantly more likely to allow vaccination of children under their care compared to parents from the richest households. On the basis of religious affiliations, Muslim parents (OR = 1.528; P<0.05) were significantly more likely to allow the vaccination of children under their care compared to Christian parents. Also, the ethnicity of parents was significantly associated with willingness to allow vaccination of children [Ewe (OR = 0.439; P<0.05) and Ga/Dangme (OR = 0.452; P<0.05)]. Moreover, Also, the region of residence was significantly associated with parents’ willingness to allow vaccination of children under their care (see Table 3).

### Results for multiple regression analysis

The multiple regression results are also presented in Table 3. Expectedly, parents with awareness of the malaria vaccine (OR = 2.200; P<0.01) were significantly more likely to allow the vaccination of children under their care compared to those without awareness of the vaccine. Also, parents from the middle-income households (OR = 9.342; P<0.05) and those from the poorest households (OR = 9.409; P<0.05) were all significantly more likely to allow the vaccination of children under their care compared to those from the richest households. With regards to ethnic backgrounds, parents from the Mande ethnic group (OR = 0.106; P<0.05) were significantly less likely to allow the vaccination of children under their care compared to parents from the Akan ethnic group. Furthermore, those who knew that the cost of vaccination is covered by the National Health Insurance (NHIS) (OR = 2.407; P<0.05) were significantly more likely to allow the vaccination of children compared to those who did not know. Geographically, parents residing in rural areas where significantly (OR = 0.254; P<0.05) less likely to allow vaccination of their children compared to those in urban areas.

## Discussion

Guided by TPB, this study examined the determinants of child malaria vaccine uptake in Ghana. The association between maternal awareness (i.e., vaccine awareness, and knowing that the vaccine is covered by NHIS), wealth, and parents’ willingness to allow vaccination of their children under-five, highlight the relevance of TPB in explaining the determinants of the child malaria vaccine uptake. The safety and efficacy of vaccines, trust in healthcare systems (Troiano and Nardi 2021), as well as socioeconomic and geographical factors may influence how parents exercise their volitional control over the vaccination of children under-five.

Consistent with earlier studies [13] our findings reiterate that having knowledge of vaccine is significantly associated with willingness to vaccinate children against malaria infection. Although parental knowledge is a major influencing factor for immunization of children under five in Africa at large [28], it is however, important to emphasize that knowing or hearing about the vaccine may not automatically translate into parents’ willingness to allow their children for vaccination against malaria. Rather, other factors including the quality or source of the information received, as well as perceptions of parents about the safety and efficacy [29] of the vaccine also influences their judgement and willingness to allow their children to get vaccinated. Thus, individual, financial, and social issues interact to influence the adoption and implementation of new health interventions such as the malaria vaccine. A similar study in Ethiopia shows that hesitance towards child vaccination against malaria is mainly due to issues related to side effects, expensiveness of the vaccine, refusal of partners [30] among others. In addition to the dire need to increase awareness creation both through media outlets, and community health education events, the malaria vaccine should be included in the Expanded Programme on Immunization (EPI). EPI has proven effective in immunization coverage. A study in Bangladesh for instance, has indicated the significant impact of the EPI programme in the country’s high coverage rates for infants and children immunization, which led to a significant reduction in child mortality rates [31]. Although the EPI programme is currently in operation in Ghana, the malaria vaccine is not part of the programme schedule. Incorporating the RTS,S/AS01 malaria vaccine into EPI would yield significant impact in preventing malaria induced child morbidities and mortalities.

The positive association between guardians’ awareness of the vaccine being covered by the National Health Insurance Scheme (NHIS), and the higher odds of willingness to allow the vaccination of children under-five, supports *TPB* emphasis that the performance and success of most health behaviors depend on nonmotivational factors including the availability of necessary opportunities and resources including knowledge, time, and skills [14]. A similar study in Ghana on guardians’ demand for children vaccination against measles and fever revealed that irrespective of the presence of cost-effective and safe vaccines, parents with health insurance were more positively associated with higher demand for vaccination of their children compared to those without NHIS [32]. Lack of knowledge on the cost of vaccines may induce hesitancy towards vaccination hence the need to increase awareness creation on the cost of vaccination.

Interestingly, parents in the poorest, and middle-income households were all positively associated with the likelihood to allow vaccination of children under-five compared to those from the richest households. This finding may be due to higher demand for malaria prevention and control measures among parents in households with poor socioeconomic statuses [33] given that such households are more likely to be living in vulnerable conditions. Thus, parents from lower socioeconomic households may have higher perceived risk of malaria infection hence the willingness to get their children vaccinated against malaria [34]. Likewise, there may exist a strong level of trust in the healthcare system among parents with lower socioeconomic status [35]. Moreover, proximity to vaccination centers, past experiences regarding time and length of waiting queues may collectively account for this observed association.

Also, ethnicity significantly predicted parents’ willingness to allow vaccination of their children against malaria, a finding that is consistent with prior studies on child vaccination in Ghana [32]. Globally, ethnicity has been reported as a major determining factor [36–39] on issues regarding vaccine uptake, with ethnic minorities usually being the least covered ethnic groups. In the Ghanaian context, both ethnic belief systems [40], and vaccine distribution biases tend to shape these disparities. This calls for the need to prioritize and target ethnic minorities (including the Mande ethnic group) in critical health matters like the new malaria vaccine program to ensure equitable coverage.

Geographically, a negative association was observed between rurality and parents’ willingness to allow vaccination of their children under-five. Urban areas tend to have more and better equipped health facilities, which in most cases are the hubs of vaccination. Even though rural parents may face greater risk of malaria infection and may be ready take preventative measures including vaccination [41], lack of proximity to designated vaccination centers, cost of transport to neighboring urban health facilities, long queues and waiting times at health facilities [42, 43], may all serve as barriers to rural parents hence the hesitance get their children vaccinated against malaria. This underscores the need to prioritize rural areas in the establishment of vaccination centers.

Although this study provides valuable insights on factors associated with parents’ willingness to allow vaccination of their children under five, it however has some noteworthy limitations. First, the study used a secondary dataset which does not capture all conceptually relevant variables including respondents’ occupation. Also, the study employed a quantitative approach for the analysis of data which limits interpretation of the findings to statistical association. Future studies could adopt a mixed method approach to unearth the nuances and perspectives of individual participants.

## Conclusions

Our study points to parental knowledge, socioeconomic status, ethnicity, and geographical location as factors that influences willingness to allow vaccination of children under five against malaria. Different contexts (i.e., ethno-cultural, socio-economic, religious, geo-political factors, etc.) exhibit varied attitudes towards mass vaccination exercises. Hence, it is crucial that health policy formulators harmonize and incorporate contextual factors into the design of preventive healthcare. The findings suggest that predischarge educational campaigns are key for improving community awareness on the relevance of vaccination, especially for uneducated parents. The cornerstones to improving vaccine uptake and fighting non-grounded fears about vaccines, is health education and correct communication of information.
